# Supplementary material for: Mutations in PCYT2 disrupt etherlipid biosynthesis and cause a complex hereditary spastic paraplegia
Source: Brain. 2019 Oct 22;142(11):3382–97. doi: 10.1093/brain/awz291 (PMC6821184; doi:10.1093/brain/awz291)
Supplement: awz291_Supplementary_Data [file awz291_supplementary_data.zip › awz291-Suppl_data/Supplementary_Data4.zip!/Supplementary information 3 - fibroblast lipidomics.zip!/Fibroblast lipidomics/Controle vs Patient1/Statistical plots and data/TG[O] species/Random forest.pdf]

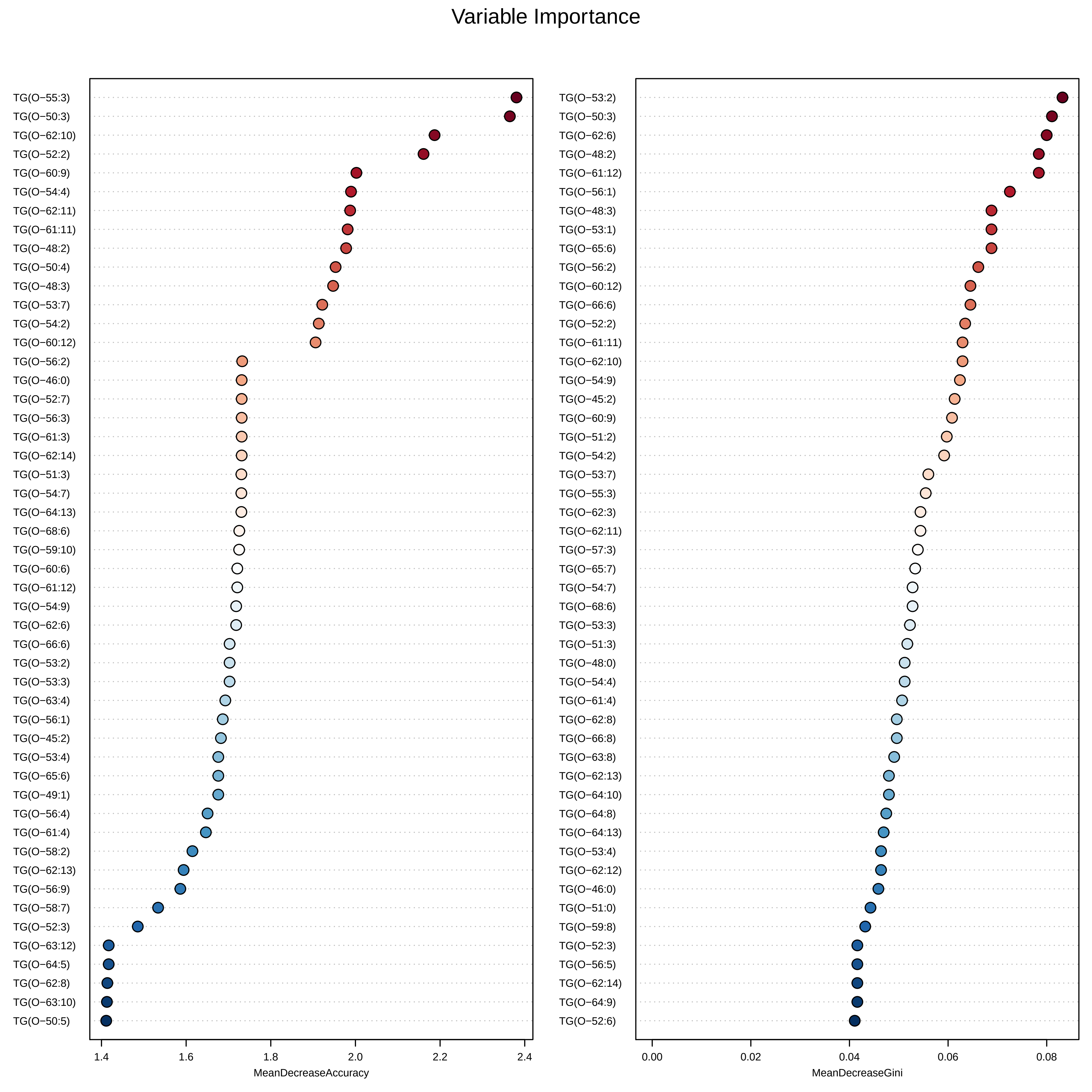

Variable Importance
TG(O−55:3)
TG(O−50:3)
TG(O−62:10)
TG(O−52:2)
TG(O−60:9)
TG(O−54:4)
TG(O−62:11)
TG(O−61:11)
TG(O−48:2)
TG(O−50:4)
TG(O−48:3)
TG(O−53:7)
TG(O−54:2)
TG(O−60:12)
TG(O−56:2)
TG(O−46:0)
TG(O−52:7)
TG(O−56:3)
TG(O−61:3)
TG(O−62:14)
TG(O−51:3)
TG(O−54:7)
TG(O−64:13)
TG(O−68:6)
TG(O−59:10)
TG(O−60:6)
TG(O−61:12)
TG(O−54:9)
TG(O−62:6)
TG(O−66:6)
TG(O−53:2)
TG(O−53:3)
TG(O−63:4)
TG(O−56:1)
TG(O−45:2)
TG(O−53:4)
TG(O−65:6)
TG(O−49:1)
TG(O−56:4)
TG(O−61:4)
TG(O−58:2)
TG(O−62:13)
TG(O−56:9)
TG(O−58:7)
TG(O−52:3)
TG(O−63:12)
TG(O−64:5)
TG(O−62:8)
TG(O−63:10)
TG(O−50:5)
1.4
1.6
1.8
2.0
2.2
2.4
MeanDecreaseAccuracy
TG(O−53:2)
TG(O−50:3)
TG(O−62:6)
TG(O−48:2)
TG(O−61:12)
TG(O−56:1)
TG(O−48:3)
TG(O−53:1)
TG(O−65:6)
TG(O−56:2)
TG(O−60:12)
TG(O−66:6)
TG(O−52:2)
TG(O−61:11)
TG(O−62:10)
TG(O−54:9)
TG(O−45:2)
TG(O−60:9)
TG(O−51:2)
TG(O−54:2)
TG(O−53:7)
TG(O−55:3)
TG(O−62:3)
TG(O−62:11)
TG(O−57:3)
TG(O−65:7)
TG(O−54:7)
TG(O−68:6)
TG(O−53:3)
TG(O−51:3)
TG(O−48:0)
TG(O−54:4)
TG(O−61:4)
TG(O−62:8)
TG(O−66:8)
TG(O−63:8)
TG(O−62:13)
TG(O−64:10)
TG(O−64:8)
TG(O−64:13)
TG(O−53:4)
TG(O−62:12)
TG(O−46:0)
TG(O−51:0)
TG(O−59:8)
TG(O−52:3)
TG(O−56:5)
TG(O−62:14)
TG(O−64:9)
TG(O−52:6)
0.00
0.02
0.04
0.06
0.08
MeanDecreaseGini
